# Socio-Economic Determinants of Care-Seeking Knowledge for Persistent Dyspeptic Symptoms in a High-Prevalence Helicobacter pylori Region

**DOI:** 10.7759/cureus.117535

**Published:** 2026-10-06

**Authors:** Edrous Alamer

**Affiliations:** 1 Department of Medical Laboratory Technology, Faculty of Nursing and Health Sciences, Jazan University, Jazan, SAU

**Keywords:** care-seeking knowledge, dyspepsia, helicobacter pylori, jazan, preventative healthcare, saudi arabia, socio-economic determinants, students

## Abstract

Background

*Helicobacter pylori* remains a public health priority in southern Saudi Arabia due to its association with gastric pathology. While previous studies have described awareness levels, few have examined the factors associated with recognizing when symptoms warrant medical care. This study identifies independent predictors of care-seeking knowledge for persistent dyspeptic symptoms among university students in a high-prevalence region.

Methods

An analytical cross-sectional study of 382 students was conducted using a questionnaire originally designed to assess *H. pylori* knowledge. The outcome was a single-best-answer item asking in which situation an infected person should receive medical intervention; selecting persistent upper abdominal pain was coded as correct. Binary logistic regression was used to model the associations of socio-economic factors and *H. pylori* knowledge with this outcome.

Results

Overall, 25.7% of students identified persistent upper abdominal pain as warranting medical intervention. *H. pylori* knowledge scores were not significantly associated with this outcome, whereas household income (>10,000 vs. <4,000 SAR; adjusted odds ratio (AOR)=2.45, p=0.007) and age (AOR=1.12 per year, p=0.003) were significant independent predictors; residing in a household of more than five members showed an exploratory association (AOR=1.88, p=0.045).

Conclusion

Recognition of when dyspeptic symptoms warrant medical care appeared to follow socio-economic and age gradients rather than general *H. pylori* knowledge. These findings should be confirmed using validated measures of care-seeking intention and actual healthcare use.

## Introduction

*Helicobacter pylori* infection remains a leading etiological factor for chronic gastritis, peptic ulcer disease, and gastric adenocarcinoma, with regional seroprevalence in Jazan Province, Saudi Arabia, estimated at approximately 47% [1,2]. While the clinical role of *H. pylori* in the pathogenesis of gastrointestinal malignancies is well established, the current clinical shift toward a comprehensive "screen-and-treat" strategy necessitates a higher standard of health literacy among the general population [3,4]. Within the framework of the Saudi Vision 2030 healthcare transformation, the national agenda has transitioned from a model of reactive clinical management toward proactive, community-based preventative care initiatives [5,6]. Despite these systemic advancements, a substantial gap persists between theoretical health awareness and its practical application in care-seeking decisions, particularly among university-aged populations who serve as the nation’s future health ambassadors [7,8].

Current global research indicates that even in regions where public health awareness regarding gastric pathogens is high, the delay between the manifestation of dyspeptic symptoms and formal clinical consultation is an important contributor to long-term disease complications [9,10]. Recent literature from 2025-2026 highlights that academic background - specifically enrollment in health-related sciences - does not necessarily translate into appropriate care-seeking [11,12]. This phenomenon suggests that socio-economic determinants, digital health literacy, and ingrained psychological barriers may outweigh formal academic training when students are faced with personal health decisions [3,7,8,12]. Furthermore, socio-economic disparities are associated with differences in care-seeking, with individuals from higher-income households often engaging earlier with diagnostic services, which may reduce the probability of disease progression [1,9].

Understanding these multidimensional predictors is imperative for developing effective, localized public health interventions that move beyond standardized educational messaging [3,4]. This study uses binary logistic regression to move beyond describing knowledge levels toward identifying factors associated with recognizing when dyspeptic symptoms warrant medical care within the Jazan University student population [11,13,14]. By identifying factors independently associated with this care-seeking knowledge, this research provides evidence to refine regional preventative strategies and optimize the early detection of *H. pylori*-associated pathologies in high-prevalence settings [10,11]. The primary objective of this study was to identify socio-economic and *H. pylori* knowledge predictors of care-seeking knowledge among university students in Jazan Province. Care-seeking knowledge was defined as recognizing persistent upper abdominal pain as a symptom requiring medical intervention..

## Materials and methods

Study design and setting

This study employed an analytical, cross-sectional design to conduct a granular investigation into the multifaceted determinants of care-seeking knowledge for persistent dyspeptic symptoms among the university student population. The study was conducted at Jazan University, located in the Jazan Province of Saudi Arabia, over a five-month duration from November 2021 to March 2022. This setting was strategically selected due to the region's documented high prevalence of *H. pylori* and the university’s status as a centralized hub for both healthcare education and diverse student demographics.

Sampling and data collection

A convenience sampling strategy was utilized to recruit 382 participants representing a wide array of academic faculties, encompassing both health-related specialties - such as pharmacy and nursing - and non-health-related fields, including engineering and science. Inclusion criteria were strictly limited to currently enrolled students aged between 18 and 30 years to maintain cohort homogeneity. Data acquisition was conducted using a structured electronic questionnaire originally designed and administered in Arabic to assess Jazan University students’ general knowledge of *H. pylori* and related diseases. The instrument was not designed to measure care-seeking intention or behavior; the present analysis uses one of its knowledge items (Section 2, Item 12) as the outcome (see the next subsection, "Variables and instrumentation"). Content validity was established by a panel of three expert academic microbiologists and public health specialists at Jazan University who evaluated item relevance, clarity, and domain coverage. A pilot pretest was conducted among 30 undergraduate students (excluded from the final N=382 sample) to evaluate semantic clarity and completion time (Cronbach’s alpha=0.78). Missing or incomplete responses accounted for <1.5% of the collected records and were excluded by listwise deletion; all analyses are based on the 382 complete responses. The questionnaire was administered in Arabic; a faithful English translation of the questionnaire exactly as administered is provided in the Appendices Table (Table 4).

Variables and instrumentation

The questionnaire (Appendix Table) provided the following study variables.

Monthly Household Income

Income was recorded using four categories (<4,000 SAR; 4,000-10,000 SAR; 10,000-20,000 SAR; >20,000 SAR). Before any regression modelling, the two highest categories were merged because of small cell sizes, giving three analytical tiers: Low Income (<4,000 SAR; reference category), Moderate Income (4,000-10,000 SAR), and High Income (>10,000 SAR). No original category was split; the only recoding was the merging of the two upper categories.

Household Size

The questionnaire offered three options (“2 or fewer," "2-5," and “more than 5” members). Because the first two options overlap at two members, categories were analyzed as recorded: Small (≤2 members; reference), Medium (2-5 members), and Large (>5 members).

Primary Source of Information

Section 1, Item 10 responses were grouped as social media, research, and reading (academic sources), and family or other sources (reference category).

H. pylori Knowledge Index

Section 2 comprises 14 single-best-answer items on *H. pylori* classification, symptoms, risk factors, transmission, diagnosis, prevention, treatment, and complications. Each correct response scored 1 point and each incorrect or unanswered response 0 points, giving a raw score of 0-14 (percentage score=(raw score/14)×100). All 14 items were weighted equally, and none were excluded. Following pre-specified academic grading benchmarks, scores were grouped into three tiers: Low Knowledge (<50%; 0-6 points; reference category), Moderate Knowledge (50-75%; 7-10 points), and High Knowledge (>75%; 11-14 points). Because Item 12 (below) has a keyed correct answer, it contributed to the 14-item index as well as defining the outcome; this overlap is addressed in the Limitations.

Outcome Variable

The outcome was derived from Section 2, Item 12, which asked (translated from Arabic), “If a person is infected, in which of the following situations should there be a health intervention?” The four response options were (1) persistent upper abdominal pain, (2) persistent headache, (3) diarrhea, and (4) weight loss in the last six months. Participants selecting option 1 were coded 1, and those selecting options 2, 3, or 4 were coded 0. This item measures knowledge of when medical care is warranted for persistent dyspeptic symptoms (hereafter “care-seeking knowledge”). It does not measure participants’ own intention to seek care, previous consultation behavior, or actual healthcare utilization.

Conceptual Distinction

The remaining Section 2 items assess *H. pylori*-specific factual knowledge, whereas Item 12 concerns symptom appraisal. Although Item 12 is framed around an infected person, persistent upper abdominal pain is a non-specific dyspeptic symptom; the outcome is therefore interpreted as recognition of when persistent dyspeptic symptoms warrant medical care, not as *H. pylori*-specific care-seeking.

Statistical analysis

To identify factors independently associated with care-seeking knowledge, a single, comprehensive multivariable binary logistic regression model was specified using the Enter method in SPSS (version 23; IBM Corp, Armonk, NY). All seven pre-specified theoretical covariates were entered simultaneously into the model: Age (continuous, per year), Sex (categorical; reference: Male), Academic Field (categorical; reference: Non-Health-Related Major), Monthly Household Income (categorical tiers: Low Income (<4,000 SAR, reference), Moderate Income (4,000-10,000 SAR), High Income (>10,000 SAR), Household Size (categorical tiers: Small (≤2 members, reference), Medium (two to five members), Large (more than five members)), Primary Source of Information (categorical tiers: Family/Other Sources [reference], Social Media, Research and Reading), and *H. pylori* Knowledge Tier (categorical tiers: Low Knowledge (<50%, reference), Moderate Knowledge (50%-75%), High Knowledge (>75%)). All reported adjusted odds ratios (AOR) and 95% confidence intervals (CI) represent mutually adjusted estimates controlling simultaneously for all other covariates in the model for the odds of selecting persistent upper abdominal pain on Item 12. The unadjusted association between knowledge tier and the outcome was assessed using Pearson’s chi-square test. Statistical significance was established at two-tailed p<0.05.

Ethical considerations

The study protocol was reviewed and granted formal ethical clearance by the Jazan University Ethics Committee under the approval reference number REC-43/05/086. The research was conducted in strict adherence to the ethical mandates of the Declaration of Helsinki and the guidelines established by the Saudi National Committee of Bioethics. To maintain the integrity of the data and protect the rights of the participants, all responses were anonymized, and digital data were encrypted to ensure strict confidentiality throughout the analytical and reporting phases.

## Results

The study cohort comprised 382 students, and their baseline demographic and socio-economic characteristics are summarized in Table 1. Most participants were aged 22-25 years (183, 47.9%), and 218 (57.1%) were female. Students from non-health-related majors accounted for 206 (53.9%) participants and those from health-related majors for 176 (46.1%). The most common monthly family income category was 4,000-10,000 SAR (158, 41.4%), and most students lived in households of more than five members (283, 74.1%). Family or other sources were the most frequently reported primary source of information about *H. pylori* (185, 48.4%), followed by social media (115, 30.1%) and research and reading (82, 21.5%). Nearly half of the students had moderate *H. pylori* knowledge (185, 48.4%), whereas 128 (33.5%) had low and 69 (18.1%) had high knowledge. Overall, 98 of the 382 participants (25.7%) selected persistent upper abdominal pain on Item 12, the study outcome. 

**Table 1:** Baseline Demographic and Socio-Economic Characteristics of the Study Cohort (n=382) Data are presented as absolute counts (n) followed by relative percentages in parentheses. All subcategories are mutually exclusive. N: Total number of participants in the study cohort; SAR: Saudi Arabian Riyal.

Variables	Category	Number of Participants (%)
Age Group	18-21 years	145 (38)
	22-25 years	183 (47.9)
	26-30 years	54 (14.1)
Sex	Male	164 (42.9)
	Female	218 (57.1)
Academic Field	Health-related major	176 (46.1)
	Non-health-related major	206 (53.9)
Monthly Family Income (SAR)	<4,000 SAR	112 (29.3)
	4,000–10,000 SAR	158 (41.4)
	>10,000 SAR	112 (29.3)
Household Size	Small (≤2 members)	27 (7.1)
	Medium (2-5 members)	72 (18.8)
	Large (>5 members)	283 (74.1)
Primary Source of Information	Social media	115 (30.1)
	Research and reading	82 (21.5)
	Family/Other sources	185 (48.4)
H. pylori Knowledge Tier	Low knowledge (<50%)	128 (33.5)
	Moderate knowledge (50–75%)	185 (48.4)
	High knowledge (>75%)	69 (18.1)

Independent predictors of care-seeking knowledge

A single multivariable binary logistic regression model containing all pre-specified sociodemographic and knowledge covariates was evaluated (Table 2). Controlling for all other factors, demographic age (AOR=1.12, 95% CI: (1.04-1.21), Wald χ²=8.84, p=0.003) and high monthly household income (>10,000 SAR vs. <4,000 SAR reference; AOR=2.45, 95% CI: (1.32-4.56), Wald χ²=7.35, p=0.007) were independently associated with care-seeking knowledge (Table 2); moderate household income (4,000-10,000 SAR) was not (AOR=1.42, 95% CI: (0.88-2.29), p=0.156). Residing in a large household (more than five members vs. two members or less, reference; AOR=1.88, 95% CI: (1.01-3.49), Wald χ²=4.02, p=0.045) was also statistically significant in the adjusted model, although the lower confidence limit was close to one. Conversely, enrollment in a health-related academic major (AOR=1.05, 95% CI: (0.82-1.34), Wald χ²=0.16, p=0.690), high *H. pylori* knowledge (>75% vs. <50% reference; AOR=1.14, 95% CI: (0.62-2.10), p=0.672), and primary information source (social media: AOR=0.85, p=0.550; research and reading: AOR=1.55, p=0.080) were not significantly associated with care-seeking knowledge (Table 2).

**Table 2:** Single Multivariable Binary Logistic Regression Model of Independent Predictors of Care-Seeking Knowledge (n=382) *Note*: Statistically significant at p<0.05. All reported adjusted odds ratios (AOR) represent mutually adjusted estimates derived simultaneously from a single multivariable binary logistic regression model controlling for all covariates in the table. Outcome: selecting persistent upper abdominal pain on Section 2, Item 12 (coded 1) vs. any other option (coded 0). Income: the two highest questionnaire categories (10,000-20,000 and >20,000 SAR) were merged. Household size categories are shown as worded in the questionnaire.

Variable / Predictor	Category / Coding	Adjusted Odds Ratio (AOR)	95% Confidence Interval	Test Statistic (Wald χ², df = 1)	p-value
Age	Per-year increase (continuous)	1.12	1.04-1.21	8.84	0.003*
Sex	Male	1.00 (Ref)	-	-	—
	Female	0.98	0.76-1.26	0.02	0.880
Academic Field	Non-health-related major	1.00 (Ref)	-	-	-
	Health-related major	1.05	0.82-1.34	0.16	0.690
Monthly Family Income	Low income (<4,000 SAR)	1.00 (Ref)	-	-	-
	Moderate income (4,000–10,000 SAR)	1.42	0.88-2.29	2.01	0.156
	High income (>10,000 SAR)	2.45	1.32-4.56	7.35	0.007*
Household Size	Small (≤2 members)	1.00 (Ref)	-	-	-
	Medium (2-5 members)	1.25	0.70-2.23	0.57	0.450
	Large (>5 members)	1.88	1.01-3.49	4.02	0.045*
Primary Information Source	Family/other sources	1.00 (Ref)	-	-	-
	Social media	0.85	0.49-1.46	0.36	0.550
	Research and reading	1.55	0.95-2.53	3.06	0.080
H. pylori Knowledge Tier	Low knowledge (<50%)	1.00 (Ref)	-	-	-
	Moderate knowledge (50-75%)	1.08	0.68-1.72	0.11	0.740
	High knowledge (>75%)	1.14	0.62-2.10	0.18	0.672

*H. pylori* knowledge and care-seeking knowledge

To examine the unadjusted relationship between *H. pylori* knowledge and care-seeking knowledge, participants were stratified across performance tiers on the 14-item *H. pylori* Knowledge Index (Table 3). Persistent upper abdominal pain was selected by 24.2% (n=31/128) of low-knowledge students, 25.9% (n=48/185) of moderate-knowledge students, and 27.5% (n=19/69) of high-knowledge students. A Pearson chi-square test showed that these proportions did not differ significantly across knowledge tiers (χ²(2)=0.27, p=0.872). This unadjusted finding aligns with our multivariable model (Table 2), indicating that general *H. pylori* knowledge was not associated with recognizing when persistent dyspeptic symptoms warrant medical care.

**Table 3:** Care-Seeking Knowledge (Section 2, Item 12) by H. pylori Knowledge Tier (n=382)

*Helicobacter pylori* knowledge tier	Other option selected (coded 0), n (%)	Persistent upper abdominal pain selected (coded 1), n (%)	Total, n (%)	Test Statistic (χ², df=2)	p-value
Low knowledge (<50%)	97 (75.8)	31 (24.2)	128 (33.5)	0.27	0.872
Moderate knowledge (50-75%)	137 (74.1)	48 (25.9)	185 (48.4)	-	-
High knowledge (>75%)	50 (72.5)	19 (27.5)	69 (18.1)	-	-
Total cohort	284 (74.3)	98 (25.7)	382 (100)	-	-

## Discussion

The findings of this study suggest a gap between general *H. pylori* knowledge and recognition of when persistent dyspeptic symptoms warrant medical care among university students in Jazan [3]. Crucially, our analytical framework distinguishes between *H. pylori*-specific factual knowledge and knowledge of when persistent dyspeptic symptoms warrant medical intervention. Because upper abdominal pain and dyspepsia are clinically non-specific symptoms that may stem from various gastrointestinal etiologies, this outcome reflects general symptom appraisal rather than *H. pylori*-specific care-seeking. While previous regional research has primarily focused on measuring baseline knowledge levels or documenting public awareness gaps [3,11], in our data, *H. pylori* knowledge - even at high levels - was not significantly associated with care-seeking knowledge in either unadjusted (χ²(2)=0.27, p=0.872; Table 3) or adjusted analysis (AOR=1.14, p=0.672) [3,10]. This observation raises questions about the assumption that expanding educational messaging will by itself reduce delayed clinical presentation and infection-related complications [3,7]. Similar disconnects between theoretical knowledge and practical healthcare utilization have been documented across Saudi Arabia and the broader Middle East, where enrollment in health sciences did not necessarily translate into timely care-seeking for upper gastrointestinal symptoms [7,12]. Instead, monthly household income (>10,000 SAR vs. <4,000 SAR; AOR=2.45, p=0.007) and age (AOR=1.12, p=0.003) emerged as independent factors associated with care-seeking knowledge (Table 2). Furthermore, residing in a large household (more than five members; AOR=1.88, p=0.045) represents a potential exploratory association that may reflect intrafamilial exposure and shared decision-making (Table 2), though this finding requires further prospective confirmation.

The identification of monthly household income (AOR=2.45, Wald χ²(1)=7.35, p=0.007) and age (AOR=1.12, Wald χ²(1)=8.84, p=0.003) as independent predictors (Table 2) suggests that socioeconomic position and age are associated with care-seeking knowledge in this setting [1,5,12]. Students from households earning >10,000 SAR per month had approximately 2.5 times the odds of identifying persistent upper abdominal pain as warranting medical intervention compared with students from households earning <4,000 SAR. One possible explanation, which we could not test, is that students from higher-income households have more prior contact with healthcare services, including private clinics, and therefore greater familiarity with which symptoms warrant evaluation. Although public healthcare services in Saudi Arabia are fully subsidized, indirect costs - such as travel from rural areas of Jazan Province or loss of academic time - may limit such contact among lower-income students [1,5,12]. These observations are consistent with reported socio-economic gradients in digestive disease outcomes in the region [9,12]. The positive association with age may reflect greater accumulated health experience or risk awareness among older students, although neither was measured in this study [10,14].

Household size was also statistically significant (AOR=1.88, Wald χ²(1)=4.02, p=0.045) (Table 2), with students from households of more than five members having higher odds of identifying persistent upper abdominal pain as warranting care. Given the borderline p-value and a lower confidence limit close to 1, this association should be regarded as exploratory. While this finding aligns with epidemiological models of intrafamilial *H. pylori* clustering in high-density environments [4,13], we emphasize that intrafamilial transmission history was not directly measured in this cohort. Exposure to symptomatic family members or shared family discussion of health is one possible explanation that warrants prospective evaluation in future studies [4,7,11,13].

In contrast, the source of health information (social media or research and reading, compared with family or other sources) was not significantly associated with care-seeking knowledge (p=0.550 and p=0.080, respectively) (Table 2) [3,8]. Although digital platforms are overwhelmingly utilized by Saudi youth for health information retrieval [8,15], our results do not indicate that the information source was associated with recognizing when symptoms warrant medical care. To align with the goals of Saudi Vision 2030, regional public health strategies may benefit from pairing awareness campaigns with accessible, low-barrier campus health services, an approach that should be evaluated in future studies [5,6].

Limitations

While this study provides insights into care-seeking knowledge among university students in a high-prevalence region, several limitations should be noted. First, the cross-sectional design establishes statistical association rather than true causality between socio-economic factors and care-seeking knowledge. Second, the reliance on convenience sampling within a single university setting may introduce selection bias, limiting the direct generalizability of these findings to non-university youth populations across Saudi Arabia. Third, and most importantly, the outcome was a single knowledge item from a questionnaire originally designed to assess *H. pylori* knowledge. It captures whether participants recognized persistent upper abdominal pain as warranting medical intervention in an infected person; it does not measure their own intention to seek care, and it may not predict actual care-seeking behavior. Because the item used a single-best-answer format, participants who selected weight loss itself as a recognized alarm feature were coded 0, which may have introduced misclassification. In addition, Item 12 contributed one point to the 14-item *H. pylori* knowledge index, so the predictor and outcome partially overlap; this would be expected to inflate rather than mask an association between knowledge and the outcome, and none was observed. Fourth, the household size options in the original questionnaire overlapped at two members, which may have caused minor misclassification between the small and medium categories. Fifth, while our model controlled for key sociodemographic factors and *H. pylori* knowledge, unmeasured confounders - such as personal health insurance status, health anxiety levels, and direct family history of gastric malignancy - were not evaluated and warrant investigation in prospective longitudinal studies. Finally, data collection was conducted between November 2021 and March 2022. While these findings provide baseline insights into student knowledge, ongoing health-system transformations under Saudi Vision 2030 may have altered current healthcare access dynamics, and these estimates should be interpreted as foundational historical benchmarks rather than current estimates for today’s students.

## Conclusions

In this sample of university students in Jazan, recognition of persistent upper abdominal pain as warranting medical intervention was associated with household income and age, but not with general *H. pylori* knowledge or academic background. This lack of association between factual knowledge and the outcome suggests that awareness campaigns alone may be insufficient to ensure that students recognize when dyspeptic symptoms need clinical evaluation. Because the outcome was a single knowledge item rather than a measure of intention or behavior, and the household size association was exploratory, these findings should be confirmed in studies using validated measures of care-seeking intention and actual healthcare utilization. In line with the goals of Saudi Vision 2030, university-based initiatives that combine symptom education with accessible, low-barrier care may help make timely evaluation more equitable across socio-economic groups.

## Appendices

Questionnaire

Study Title: Measuring the Knowledge of Jazan University Students about *Helicobacter pylori* (Stomach Bacterium).

Study Objective: This study aims to measure the level of general knowledge among Jazan University students regarding *H. pylori* and its related diseases.

Confidentiality: All data are strictly confidential and accessed solely by the research team.

Consent and Withdrawal: Participation is entirely voluntary; participants may withdraw at any point without penalty.

Completion Time: Less than 5 minutes.

**Table 4:** English Translation of the Questionnaire as Administered to Participants *Note: Section 2, Item 12 (“If a person is infected, in which of the following situations should there be a health intervention?”) generates the binary outcome variable (care-seeking knowledge for persistent dyspeptic symptoms). Selecting “Persistent upper abdominal pain” was coded 1; selecting “Persistent headache," "Diarrhea," or “Weight loss in the last 6 months” was coded 0. All 14 items in Section 2, including Item 12, generate the *H. pylori* Knowledge Index (1 point per correct answer; range 0–14), operationalized as Low (<50%; 0-6 points), Moderate (50-75%; 7-10 points), and High (>75%; 11-14 points). Section 1, Item 5 income categories are shown as administered; the two highest categories were merged for analysis. Household size options (Section 1, Item 4) are shown as administered and overlap at two members. Minor wording in this translation has been aligned more closely with the Arabic original; no items were added, removed, or changed in meaning.

Item No.	Item / Parameter	Response Options
Section 1: General Data & Demographics		
1	Age	Years ( )
2	Sex	Male ( )
		Female ( )
3	Academic Specialty	Health-Related Major ( )
		Non-Health-Related Major ( )
4	Household Size	≤ 2 members ( )
		2–5 members ( )
		> 5 members ( )
5	Monthly Household Income (SAR)	Less than 4,000 SAR ( )
		4,000–10,000 SAR ( )
		10,000–20,000 SAR ( )
		More than 20,000 SAR ( )
6	Do you drink coffee or tea?	Yes ( )
		No ( )
7	If yes, how many cups of coffee/tea do you drink daily?	Do not drink ( )
		< 2 cups ( )
		2–5 cups ( )
		> 5 cups ( )
8	Primary drinking water source at home	Desalinated tap water ( )
		Bottled water ( )
		Both ( )
9	Have you ever heard of *Helicobacter pylori* (*H. pylori*)?	Yes ( )
		No ( )
10	If yes, from which source did you hear about it?	Family ( )
		Social Media ( )
		Research and reading ( )
		Other Sources ( )
		Never heard of it ( )
11	Have you previously been infected with *H. pylori*?	Yes ( )
		No ( )
12	If yes, were you treated for it?	Yes ( )
		No ( )
		Never infected ( )
Section 2: Knowledge about *H. pylori*		
1	*H. pylori* is usually present in the stomach in the form of:	Bacteria ( )
		Virus ( )
		Parasite ( )
		Fungus ( )
2	Which of the following is considered a primary symptom of *H. pylori* infection?	Upper abdominal pain ( )
		Increased salivation ( )
		Constipation ( )
		Lower abdominal pain ( )
3	Which of the following clinical signs may manifest in an infected individual?	Nausea ( )
		Abdominal skin rash ( )
		Hair loss ( )
		Indigestion (Dyspepsia) ( )
4	Which of the following is a recognized risk factor facilitating *H. pylori* infection?	Parasitic co-infection ( )
		Low socioeconomic status ( )
		Obesity ( )
		Household member infected ( )
5	Which of the following is NOT considered a contributing risk factor for *H. pylori* infection?	Meat consumption ( )
		Spicy/pepper-rich food ( )
		Fast food consumption ( )
		Unfiltered water ( )
6	Person-to-person transmission of *H. pylori* primarily occurs via:	Coughing and sneezing ( )
		Blood transfusion ( )
		Oral route ( )
		Houseflies ( )
7	Clinical diagnosis of *H. pylori* is established through:	Urine test ( )
		Breath test ( )
		Blood test ( )
		Saliva test ( )
8	Which of the following measures effectively aids in preventing *H. pylori* infection?	Hand hygiene (handwashing) ( )
		Vaccination ( )
		Wearing face masks ( )
		Vitamin supplementation ( )
9	Standard clinical management/treatment for an infected individual consists of:	Tablets (oral medication) ( )
		Injections ( )
		Intravenous fluids ( )
		Blood transfusion ( )
10	Standard duration of eradication therapy for *H. pylori*:	Less than 1 week ( )
		1 to 2 weeks ( )
		3 to 4 weeks ( )
		6 weeks ( )
11	To avoid infection, a person should:	Adhere to the treatment plan.
		Drink plenty of fluids.
		Take a vaccine ( )
		Avoid fatty foods.
12	If a person is infected, in which of the following situations should there be a health intervention?*	Persistent upper abdominal pain ( )
		Persistent headache
		Diarrhea ( )
		Weight loss in the last 6 months ( )
13	Regarding *H. pylori*, which of the following is correct?	Some people do not show symptoms.
		It is a serious infection.
		An acute illness lasting only a short time ( )
		Affects all body systems
14	Which of the following complications is considered a result of *H. pylori* infection?	Dehydration ( )
		Colon cancer ( )
		Gastric cancer ( )
		Viral hepatitis ( )
